# Association between dipeptidyl peptidase-4 inhibitor use and risk of Parkinson’s disease among patients with diabetes mellitus: a retrospective cohort study

**DOI:** 10.18632/aging.206074

**Published:** 2024-08-22

**Authors:** Kuang-Hua Huang, Yih Yang, Shuo-Yan Gau, Tung-Han Tsai, Chien-Ying Lee

**Affiliations:** 1 Department of Health Services Administration, China Medical University, Taichung 406040, Taiwan; 2 Department of Surgery, E-Da Hospital, I-Shou University, Kaohsiung 82445, Taiwan; 3 School of Medicine, Chung Shan Medical University, Taichung 40201, Taiwan; 4 Department of Pharmacology, Chung Shan Medical University, Taichung 40201, Taiwan; 5 Department of Pharmacy, Chung Shan Medical University Hospital, Taichung 40201, Taiwan

**Keywords:** Parkinson disease, DPP-4 inhibitors, diabetes mellitus, defined daily dose

## Abstract

Background: How a person’s Parkinson disease (PD) risk is affected by dipeptidyl peptidase-4 (DPP-4) inhibitors remains unclear. We evaluated the association of PD risk with use of these inhibitors in individuals diagnosed as having diabetes mellitus (DM).

Methods: Individuals diagnosed as having new-onset DM were enrolled into the case group and comparison group, comprising patients who received a DPP-4 inhibitor and a sulfonylurea, respectively. These groups were matched through propensity score matching on the basis of income level, gender, urbanization level, enrollment year, age, and diabetes complications severity index score. The case group was divided into subgroups on the basis of whether they had a cumulative defined daily dose (cDDD) of <75, 75–150, or >150. The DPP-4 inhibitor–PD risk association was evaluated through a Cox proportional hazards model. The Bonferroni adjustment test was employed to adjust *P*-values and reduce the false positive rate.

Results: Compared with those in the comparison group (treatment with a sulfonylurea), patients with a DPP-4 inhibitor cDDD of >150 had a hazard ratio (HR) of 1.30 for PD development (95% confidence interval [CI]: 0.97-1.73; adjusted *P* = .263); the HRs for patients with a cDDD of <75 or 75–150 were 0.95 (95% CI: 0.71-1.27; adjusted *P* = .886) and 1.06 (95% CI: 0.75-1.50; adjusted *P* = .886), respectively. We noted nonsignificant differences regarding the associations between the use of the various DPP-4 inhibitors (linagliptin, saxagliptin, sitagliptin, and vildagliptin) and PD risk after adjustment for any individual inhibitor (adjusted *P* > .05).

Conclusions: DPP-4 inhibitors were discovered in this study to not be associated with increased PD risk. This result was confirmed when the analysis was conducted individually for the 4 investigated DPP-4 inhibitors (sitagliptin, saxagliptin, linagliptin, and vildagliptin).

## INTRODUCTION

The chronic neurodegenerative ailment Parkinson disease (PD) mainly affects the systems that control an individual’s motor abilities; its mechanism involves the progressive loss of dopaminergic neurons, and it may be accompanied by cognitive and behavioral *disorders* [1]. PD risk increases with age, and this disease is, among age-related neurodegenerative motor disorders, the second most common; its prevalence in the population aged 60 years or older is 1% [2]. Alpha-synuclein represents an essential presynaptic neuronal protein linked to PD pathology. Abnormal accumulation and aggregation of alpha-synuclein are the cause of not only the neuropathological hallmark of PD but also dementia with Lewy bodies and other alpha-synuclein-related neurodegenerative disorders [3, 4].

In epidemiological studies, increases in PD risk have been attributed to diabetes mellitus (DM) [5–7]. PD risk in individuals with DM was discovered in a meta-analysis of population-based cohort studies to be 38% higher on average than that in individuals without DM [8]. Observational studies have supported this finding of increased PD risk among individuals having DM, in addition to revealing similar dysregulated pathways in DM and PD [5, 9]. Furthermore, evidence of associations of insulin resistance pathogenesis with dementia and PD is accumulating [10]. Protein misfolding, aggregation, and accumulation were discovered in many neurodegenerative diseases and were reported to potentially contribute to loss of synaptic connections, damage to neurons, and the development of neurological disorders [11]. An animal study indicated that in neuronal Lewy bodies, alpha-synuclein and tau may coaggregate, and this coaggregation may be connected to tauopathy in patients with PD [12]. However, in PD and concomitant dementia and PD, tauopathies have only been noted in the nigrostriatal dopaminergic neuronal region [13]. Tauopathy, a key characteristic of various human neurodegenerative diseases (e.g., Alzheimer’s disease and PD), is the abnormal cytoplasmic accumulation of tau protein or neurofibrillary tangles [14, 15].

Dipeptidyl peptidase-4 (DPP-4) inhibitors, which are typically orally administered, constitute a promising treatment for DM [16]. Commonly administered to individuals with DM, these inhibitors prevent glucose-dependent insulinotropic peptide (GIP) and glucagon-like peptide-1 (GLP-1) from degrading; they thus increase the levels of active hormones, which results in increased glucose-dependent insulin secretion [17]. Moreover, these inhibitors, as documented in several *in vitro* and animal studies, may possess neuroprotective effects [18, 19]. Surprisingly, an animal study found that tau phosphorylation was increased by the DPP-4 inhibitor sitagliptin, which suggested increased insulin resistance within the brain and that this drug may consequently exacerbate the symptoms of PD [20]. However, whether these preclinical findings are relevant to patients with DM who receive DPP-4 inhibitors to slow PD progression remains unknown. Whether a patient’s PD risk is increased by their use of a DPP-4 inhibitor must be urgently clarified through in-depth research, and a drug-by-drug evaluation of the mechanisms that underpin DPP-4 inhibitors is required. Accordingly, in the present retrospective cohort study, we assessed the possibility of DPP-4 inhibitor use being associated with an increased likelihood of developing PD. In addition, through nationwide-database-derived data, we investigated whether DPP-4 inhibitor use has a dose-dependent PD-risk-increasing effect in Taiwanese patients with DM.

## MATERIALS AND METHODS

### Data sources

Taiwan’s Health and Welfare Data Science Center (HWDC) has released the Longitudinal Health Insurance Database (LHID) for the period 2008–2016, and this database constituted our secondary data analysis’s data source. The LHID contains information on the beneficiaries of Taiwan’s National Health Insurance (NHI) program, and real-world evidence has been obtained using this information and employed to support clinical and healthcare policy-making [21, 22]. Since 1995, the government-run NHI has been a single-payer national social insurance program. Of Taiwan’s 23 million residents, the NHI covers >99%; in addition, of all healthcare facilities in Taiwan, >93% are contracted with the National Health Insurance Administration. Using data from the LHID, we evaluated PD risk in patients with DM who were prescribed DPP-4 inhibitors. The LHID’s data are anonymous, and when data from the LHID are requested, for privacy protection, each patient is assigned a randomized, scrambled identification number by the HWDC. This thus obviated the need for informed consent in our executed study.

### Study participants

This study enrolled individuals aged 50 years or older and who were given a first diagnosis of DM in the period 2009–2013. A patient was considered to have DM if they received a DM diagnosis thrice a year that was based on code 250 of the International Classification of Diseases, Ninth Revision, Clinical Modification (ICD-9-CM). On this basis, we included 793,802 patients with new-onset DM from 2009 to 2013. We excluded patients with the following characteristics to prevent bias: diagnosis of PD before their diagnosis of DM, diagnosis of PD within 1 year of receiving a DM diagnosis, no prescription of a sulfonylurea or DPP-4 inhibitor, and type 1 DM.

**Figure 1:**
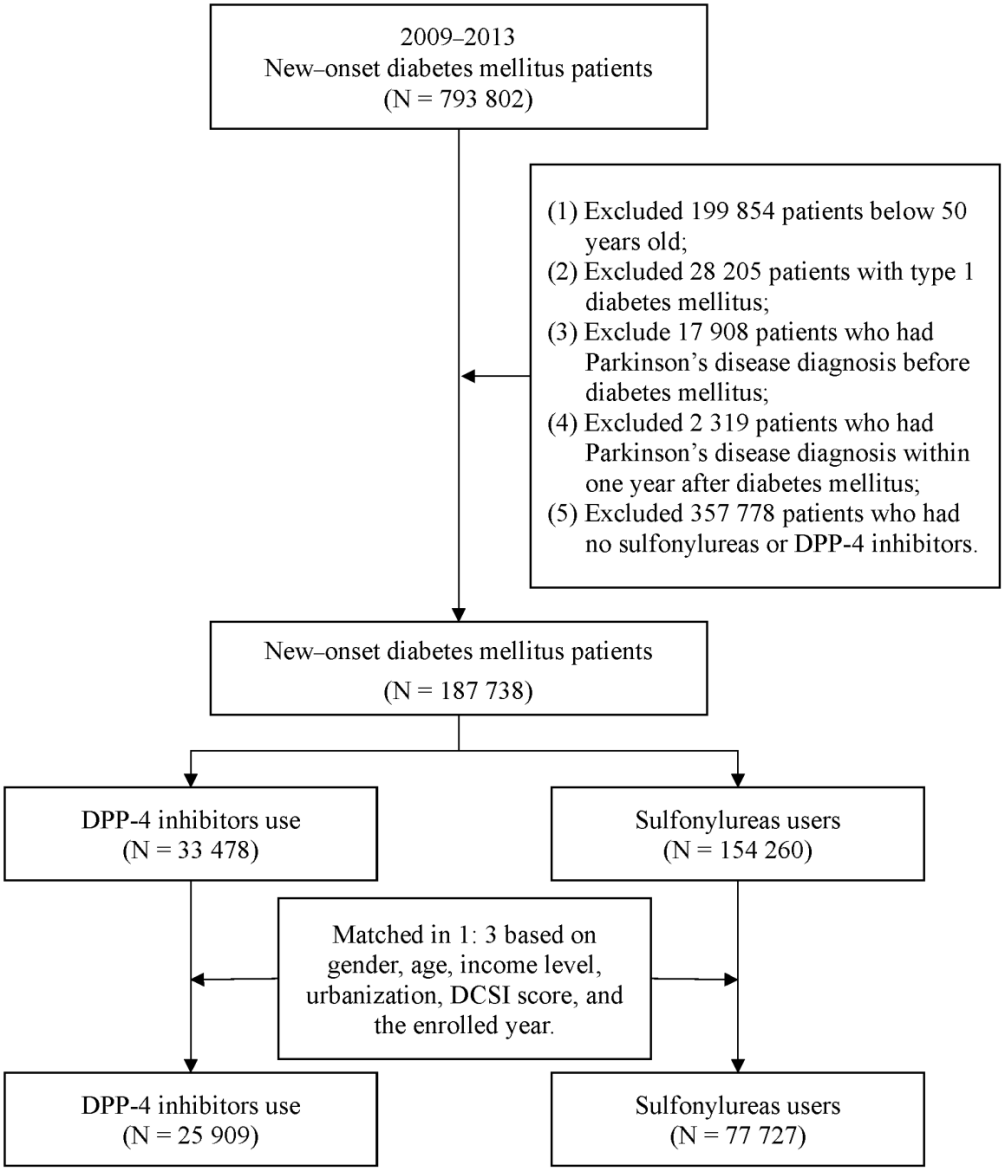
Patient selection process.

We established our case group to comprise enrolled individuals who were prescribed a DPP-4 inhibitor within the first year following their DM diagnosis. We defined DPP-4 inhibitors as outlined in the Anatomical Therapeutic Chemical (ATC) classification system and considered the following inhibitors: vildagliptin, sitagliptin, saxagliptin, and linagliptin (A10BH02, A10BH01, A10BH03, and A10BH05, respectively). To avoid selection bias, we obtained a comparison group, comprising patients who received a sulfonylurea (ATC code A10BB), through propensity score matching. Each patient who received a DPP-4 inhibitor was matched with 3 patients who received a sulfonylurea on the basis of income level, gender, urbanization level, enrollment year, age, and diabetes complications severity index (DCSI) score. After matching, the case and comparison groups comprised 25,909 and 77,727 patients, respectively. We present in Figure 1 the procedure we followed in our patient selection and enrollment.

### Study design

Our executed cohort study investigated the PD risk of the case group. DPP-4 inhibitor intake and exposure were measured using a standard approach—the defined daily dose (DDD). The DDD in adults can be considered as the assumed daily average maintenance dose, according to the World Health Organization. The cumulative DDD (cDDD) in the first post-DM-diagnosis year was calculated to reflect the patients’ DPP-4 inhibitor exposure. We referred to the relevant article on cDDDs [23] and set the medication grade interval in accordance with the actual usage of those taking a DPP-4 inhibitor. To determine whether higher cumulative use, as defined by the cDDD, was significantly associated with PD risk, the case patients were split into 3 groups, namely those with a cDDD of <75, 75–150, and >150, and we separately estimated the risk in each of these groups. In the case group, the observation start date was taken as the date of the first DPP-4 inhibitor prescription, whereas in the comparison group, it was the date of the first sulfonylurea prescription. The included patients were regarded as having been continuously exposed to their relevant drug (i.e., DPP-4 inhibitor or sulfonylurea) from 2009 to 2013. Each of the aforementioned groups was followed from the observation start date until death, use of a different drug of interest (i.e., DPP-4 inhibitor or sulfonylurea), PD diagnosis, or the end of the observation period, whichever occurred first. PD was considered to have been diagnosed if ICD-9-CM code 332 or International Classification of Diseases, Tenth Revision, Clinical Modification (ICD-10-CM) code G20 was recorded for a patient in 3 or more of their outpatient visits within a 1-year period. We followed all the enrollees from the date of DM diagnosis until death, PD diagnosis, or the end of the observation period, whichever occurred first.

The adjusted variables included the patients’ baseline characteristics, DCSI score, and comorbidities. The DCSI score was calculated from a patient’s diabetes complication status for 1 year after their DM diagnosis, and medical records from 1 year before the DM diagnosis were used to assess the following comorbidities: hyperlipidemia (ICD-9-CM 272.0–272.4), obesity (ICD-9-CM 278.00), hyperuricemia (ICD-9-CM 790.6), hypertension (ICD-9-CM 401–405), chronic kidney disease (CKD; ICD-9-CM 585), coronary artery disease (CAD; ICD-9-CM 414.0), cerebrovascular disease (CVD; ICD-9-CM 430–438), arrhythmia (ICD-9-CM 427), heart failure (ICD-9-CM 428.0), depression (ICD-9-CM 311), and anxiety (ICD-9-CM 300.0).

### Statistical analysis

We evaluated the DPP-4 inhibitor–PD risk association by employing a Cox proportional hazards model after adjustment for all relevant variables. We also derived hazard ratios (HRs) and their corresponding 95% confidence intervals (CIs), which are presented herein. We employed the Bonferroni adjustment test to reduce the false positive rate. Finally, we executed a subgroup analysis involving the subgroups of patients receiving vildagliptin, sitagliptin, saxagliptin, or linagliptin. We executed all statistical analyses, with *P* < .05 indicating statistical significance, by employing version 9.4 of SAS software (SAS Institute, Cary, NC, USA).

## RESULTS

The distribution of patient characteristics is presented in Table 1. In the case and comparison (DPP-4 inhibitor and sulfonylurea, respectively) groups, the mean ages were 61.94 ± 9.20 and 61.98 ± 9.05 years, respectively. Of the patients who received a DPP-4 inhibitor, the female proportion and male proportion were 45.34% and 54.66%, respectively. We noted between-group similarity regarding the matched variables (gender, age, income level, urbanization level, and DCSI score; *P* > .05). Of the patients who received a DPP-4 inhibitor, 41.90% had hypertension, 17.32% had hyperlipidemia, 8.38% had CAD, 4.90% had arrhythmia, 3.08% had heart failure, 1.75% had CKD, and 0.49% had obesity.

**Table 1:** Baseline characteristics of the matched case and comparison groups.

**Variables**	**Sulfonylureas**		**DPP-4**								**p-value**
			**Subtotal**		**cDDD <75**		**cDDD 75-150**		**cDDD >150**		
	**N**	**%**	**N**	**%**	**N**	**%**	**N**	**%**	**N**	**%**	
Total	77,727	100.00	25,909	100.00	10,733	100.00	6,495	100.00	8,681	100.00	
Gender											0.743
Female	35,332	45.46	11,747	45.34	4,891	45.57	2,940	45.27	3,916	45.11	
Male	42,395	54.54	14,162	54.66	5,842	54.43	3,555	54.73	4,765	54.89	
Age (year)											0.560
50-64	52,045	66.96	17,355	66.98	7,074	65.91	4,413	67.94	5,868	67.60	
65-74	16,290	20.96	5,481	21.15	2,287	21.31	1,353	20.83	1,841	21.21	
≥75	9,392	12.08	3,073	11.86	1,372	12.78	729	11.22	972	11.20	
Mean ± SD	61.98 ± 9.05		61.94 ± 9.20		62.23 ± 9.35		61.71 ± 9.13		61.74 ± 9.06		
Income level											0.702
≤21,000	30,216	38.87	10,020	38.67	4,210	39.22	2,417	37.21	3,393	39.09	
21,001-33,000	27,928	35.93	9,384	36.22	4,069	37.91	2,408	37.07	2,907	33.49	
≥33,001	19,583	25.19	6,505	25.11	2,454	22.86	1,670	25.71	2,381	27.43	
Urbanization											0.954
Level 1	20,869	26.85	7,041	27.18	2,704	25.19	1,797	27.67	2,540	29.26	
Level 2	24,674	31.74	8,154	31.47	3,229	30.08	2,041	31.42	2,884	33.22	
Level 3	12,874	16.56	4,252	16.41	1,864	17.37	1,049	16.15	1,339	15.42	
Level 4	10,960	14.10	3,672	14.17	1,609	14.99	933	14.36	1,130	13.02	
Level 5	1,706	2.19	571	2.20	253	2.36	144	2.22	174	2.00	
Level 6	3,494	4.50	1,167	4.50	582	5.42	278	4.28	307	3.54	
Level 7	3,150	4.05	1,052	4.06	492	4.58	253	3.90	307	3.54	
DCSI score											0.932
0	43,017	55.34	14,340	55.35	5,904	55.01	3,600	55.43	4,836	55.71	
1	14,485	18.64	4,805	18.55	1,923	17.92	1,247	19.20	1,635	18.83	
≥2	20,225	26.02	6,764	26.11	2,906	27.08	1,648	25.37	2,210	25.46	
Enrolled year											1.000
2009	9,663	12.43	3,221	12.43	1,256	11.70	814	12.53	1,151	13.26	
2010	14,247	18.33	4,749	18.33	1,768	16.47	1,162	17.89	1,819	20.95	
2011	18,612	23.95	6,204	23.95	2,629	24.49	1,495	23.02	2,080	23.96	
2012	18,075	23.25	6,025	23.25	2,649	24.68	1,579	24.31	1,797	20.70	
2013	17,130	22.04	5,710	22.04	2,431	22.65	1,445	22.25	1,834	21.13	
Hypertension											<0.001
No	43,826	56.38	15,053	58.10	6,349	59.15	3,697	56.92	5,007	57.68	
Yes	33,901	43.62	10,856	41.90	4,384	40.85	2,798	43.08	3,674	42.32	
Hyperlipidemia											<0.001
No	65,775	84.62	21,422	82.68	8,984	83.70	5,305	81.68	7,133	82.17	
Yes	11,952	15.38	4,487	17.32	1,749	16.30	1,190	18.32	1,548	17.83	
Hyperuricemia											0.043
No	76,995	99.06	25,628	98.92	10,619	98.94	6,420	98.85	8,589	98.94	
Yes	732	0.94	281	1.08	114	1.06	75	1.15	92	1.06	
Cerebrovascular disease											0.392
No	73,124	94.08	24,337	93.93	10,089	94.00	6,071	93.47	8,177	94.19	
Yes	4,603	5.92	1,572	6.07	644	6.00	424	6.53	504	5.81	
Coronary artery disease											<0.001
No	71,855	92.45	23,738	91.62	9,837	91.65	5,951	91.62	7,950	91.58	
Yes	5,872	7.55	2,171	8.38	896	8.35	544	8.38	731	8.42	
Arrhythmia											<0.001
No	74,751	96.17	24,639	95.10	10,213	95.16	6,154	94.75	8,272	95.29	
Yes	2,976	3.83	1,270	4.90	520	4.84	341	5.25	409	4.71	
Heart failure											<0.001
No	75,830	97.56	25,110	96.92	10,401	96.91	6,279	96.67	8,430	97.11	
Yes	1,897	2.44	799	3.08	332	3.09	216	3.33	251	2.89	
Anxiety											0.887
No	71,802	92.38	23,941	92.40	9,913	92.36	5,958	91.73	8,070	92.96	
Yes	5,925	7.62	1,968	7.60	820	7.64	537	8.27	611	7.04	
Depression											0.661
No	77,307	99.46	25,763	99.44	10,666	99.38	6,453	99.35	8,644	99.57	
Yes	420	0.54	146	0.56	67	0.62	42	0.65	37	0.43	
Chronic kidney disease											<0.001
No	76,989	99.05	25,456	98.25	10,498	97.81	6,385	98.31	8,573	98.76	
Yes	738	0.95	453	1.75	235	2.19	110	1.69	108	1.24	
Obesity											<0.001
No	77,517	99.73	25,782	99.51	10,686	99.56	6,462	99.49	8,634	99.46	
Yes	210	0.27	127	0.49	47	0.44	33	0.51	47	0.54	

We detail in Table 2 the observed incidence of PD within 3 years of DM diagnosis. Overall, PD developed in 524 patients (0.51%) in the 3 years after they received a DM diagnosis. The incidence of PD at 3 years in the patients receiving a sulfonylurea was 0.49%, whereas that in those receiving a DPP-4 inhibitor was 0.49%, 0.52%, and 0.61% for patients with a cDDD of <75, 75–150, and >150, respectively. We adjusted for related variables and then determined that compared with the patients receiving a sulfonylurea, the patients who had a DPP-4 inhibitor cDDD of <75, 75–150, and >150 had HRs of 0.95 (95% CI: 0.71-1.27; adjusted *P* = .886), 1.06 (95% CI: 0.75-1.50; adjusted *P* = .886), and 1.30 (95% CI: 0.97-1.73; adjusted *P* = .886), respectively, for developing PD.

**Table 2:** Risk of incident PD in 3-year follow-up.

**Variables**	**Parkinson’s disease**								
	**Without**		**With**		**Unadjusted model**		**Adjusted model**		
	**N**	**%**	**N**	**%**	**HR (95% CI)**	**p-value**	**HR (95% CI)**	**p-value**	**Adjusted** **p-value^†^**
Patient group									
Sulfonylureas use	77,343	99.51	384	0.49	Reference		Reference		
DPP-4 use									
cDDD <75	10,680	99.51	53	0.49	1.00 (0.75-1.33)	0.998	0.95 (0.71-1.27)	0.728	0.886
cDDD 75-150	6,461	99.48	34	0.52	1.06 (0.75-1.50)	0.748	1.06 (0.75-1.50)	0.750	0.886
cDDD >150	8,628	99.39	53	0.61	1.24 (0.93-1.65)	0.147	1.30 (0.97-1.73)	0.078	0.263
cDDD (Mean ± SD)	135.92 ± 112.20		147.06 ± 113.13		1.01 (0.99-1.02)	0.135	1.01 (0.99-1.02)	0.070	0.220
Gender									
Female	46,838	99.49	241	0.51	Reference		Reference		
Male	56,274	99.50	283	0.50	0.98 (0.82-1.16)	0.795	1.13 (0.95-1.35)	0.166	0.359
Age (year)									
50-64	69,265	99.81	135	0.19	Reference		Reference		
65-74	21,583	99.14	188	0.86	4.45 (3.57-5.56)	<0.001	3.82 (3.04-4.79)	<0.001	<0.001
≥75	12,264	98.39	201	1.61	8.35 (6.71-10.38)	<0.001	6.40 (5.06-8.11)	<0.001	<0.001
Income level									
≤21,000	40,008	99.43	228	0.57	Reference		Reference		
21,001-33,000	37,098	99.43	214	0.57	1.01 (0.84-1.22)	0.898	1.13 (0.92-1.40)	0.249	0.589
≥33,001	26,006	99.69	82	0.31	0.55 (0.43-0.71)	<0.001	0.87 (0.67-1.13)	0.304	0.438
Urbanization									
Level 1	27,803	99.62	107	0.38	Reference		Reference		
Level 2	32,671	99.52	157	0.48	1.25 (0.98-1.60)	0.077	1.21 (0.94-1.55)	0.134	0.359
Level 3	17,059	99.61	67	0.39	1.02 (0.75-1.39)	0.895	0.93 (0.68-1.26)	0.623	0.778
Level 4	14,534	99.33	98	0.67	1.75 (1.33-2.30)	<0.001	1.23 (0.93-1.63)	0.149	0.359
Level 5	2,250	98.81	27	1.19	3.10 (2.03-4.73)	<0.001	1.80 (1.17-2.76)	0.008	0.037
Level 6	4,613	98.97	48	1.03	2.69 (1.92-3.79)	<0.001	1.89 (1.33-2.69)	<0.001	0.002
Level 7	4,182	99.52	20	0.48	1.24 (0.77-2.00)	0.375	0.86 (0.53-1.39)	0.530	0.739
DCSI score									
0	57,186	99.70	171	0.30	Reference		Reference		
1	19,189	99.48	101	0.52	1.76 (1.38-2.25)	<0.001	1.39 (1.08-1.80)	0.012	0.039
≥2	26,737	99.07	252	0.93	3.14 (2.59-3.82)	<0.001	1.71 (1.36-2.15)	<0.001	<0.001
Enrolled year									
2009	12,808	99.41	76	0.59	Reference		Reference		
2010	18,891	99.45	105	0.55	0.94 (0.70-1.26)	0.665	0.89 (0.67-1.20)	0.455	0.679
2011	24,684	99.47	132	0.53	0.90 (0.68-1.20)	0.471	0.85 (0.63-1.15)	0.288	0.444
2012	23,984	99.52	116	0.48	0.82 (0.61-1.09)	0.166	0.90 (0.66-1.22)	0.482	0.691
2013	22,745	99.58	95	0.42	0.70 (0.52-0.95)	0.023	0.84 (0.61-1.15)	0.275	0.303
Hypertension									
No	58,653	99.62	226	0.38	Reference		Reference		
Yes	44,459	99.33	298	0.67	1.74 (1.46-2.07)	<0.001	0.99 (0.82-1.19)	0.896	0.895
Hyperlipidemia									
No	86,778	99.52	419	0.48	Reference		Reference		
Yes	16,334	99.36	105	0.64	1.33 (1.07-1.65)	0.009	1.04 (0.83-1.30)	0.747	0.886
Hyperuricemia									
No	102,107	99.50	516	0.50	Reference		Reference		
Yes	1,005	99.21	8	0.79	1.57 (0.78-3.16)	0.204	1.05 (0.52-2.12)	0.888	0.895
Cerebrovascular disease									
No	97,012	99.54	449	0.46	Reference		Reference		
Yes	6,100	98.79	75	1.21	2.65 (2.07-3.38)	<0.001	1.16 (0.89-1.51)	0.262	0.438
Coronary artery disease									
No	95,146	99.53	447	0.47	Reference		Reference		
Yes	7,966	99.04	77	0.96	2.05 (1.61-2.61)	<0.001	1.02 (0.78-1.32)	0.890	0.895
Arrhythmia									
No	98,913	99.52	477	0.48	Reference		Reference		
Yes	4,199	98.89	47	1.11	2.31 (1.71-3.12)	<0.001	1.12 (0.82-1.53)	0.487	0.739
Heart failure									
No	100,452	99.52	488	0.48	Reference		Reference		
Yes	2,660	98.66	36	1.34	2.77 (1.98-3.89)	<0.001	1.05 (0.73-1.51)	0.795	0.886
Anxiety									
No	95,299	99.54	444	0.46	Reference		Reference		
Yes	7,813	98.99	80	1.01	2.19 (1.73-2.78)	<0.001	1.78 (1.39-2.27)	<0.001	<0.001
Depression									
No	102,553	99.50	517	0.50	Reference		Reference		
Yes	559	98.76	7	1.24	2.47 (1.17-5.21)	0.017	1.71 (0.81-3.65)	0.162	0.359
Chronic kidney disease									
No	101,940	99.51	505	0.49	Reference		Reference		
Yes	1,172	98.40	19	1.60	3.26 (2.06-5.15)	<0.001	1.40 (0.88-2.25)	0.160	0.359
Obesity									
No	102,778	99.50	521	0.50	Reference		Reference		
Yes	334	99.11	3	0.89	1.77 (0.57-5.49)	0.326	2.07 (0.66-6.45)	0.211	0.405

^†^Adjusted p-value obtained through the Bonferroni adjustment test.

In the adjusted model, the HRs for developing PD were 3.82 (95% CI: 3.04-4.97; adjusted *P* < .001) and 6.40 (95% CI: 5.06-8.11; adjusted *P* < .001) for patients with DM aged 65–74 and ≥75 years, respectively, and were 1.39 (95% CI: 1.08-1.80; adjusted *P* = .039) and 1.71 (95% CI: 1.36-2.15; adjusted *P* < .001) for patients with DM with a DCSI score of 1 and ≥2, respectively. We observed patients with comorbid anxiety (HR: 1.78, 95% CI: 1.39-2.27; adjusted *P* < .001) to exhibit a relatively high PD risk.

We list in Table 3 the derived results of our subgroup analysis executed for subgroups defined on the basis of specific DPP-4 inhibitors. After adjustment for related variables, we did not observe the patients using linagliptin, saxagliptin, sitagliptin, or vildagliptin to exhibit a significantly increased PD risk when contrasted against those prescribed a sulfonylurea (adjusted *P* > .05).

**Table 3:** Risk of incident PD in 3-year follow-up for cDDD-based subgroups.

**Variables**	**Unadjusted model**		**Adjusted model**		
	**HR (95% CI)**	**p-value**	**HR (95% CI)**	**p-value**	**Adjusted p-value ^†^**
DPP-4 use (vs. Sulfonylurea use)					
Linagliptin					
cDDD <75	0.70 (0.18-2.81)	0.616	0.76 (0.19-3.06)	0.700	0.868
cDDD 75-150	0.46 (0.07-3.26)	0.437	0.54 (0.08-3.89)	0.544	0.752
cDDD >150	0.84 (0.21-3.37)	0.807	0.94 (0.23-3.79)	0.930	0.895
cDDD as continuous variable	1.00 (0.99-1.01)	0.780	1.00 (0.99-1.01)	0.961	0.911
Saxagliptin					
cDDD <75	0.75 (0.31-1.80)	0.518	0.78 (0.32-1.89)	0.580	0.732
cDDD 75-150	0.67 (0.22-2.07)	0.483	0.69 (0.22-2.14)	0.517	0.722
cDDD >150	0.83 (0.31-2.22)	0.709	0.94 (0.35-2.52)	0.897	0.881
cDDD as continuous variable	0.99 (0.98-1.02)	0.523	1.00 (0.99-1.01)	0.694	0.819
Sitagliptin					
cDDD <75	1.14 (0.82-1.57)	0.439	1.02 (0.73-1.41)	0.928	0.905
cDDD 75-150	1.20 (0.82-1.77)	0.343	1.16 (0.79-1.70)	0.450	0.642
cDDD >150	1.34 (0.99-1.82)	0.059	1.37 (1.01-1.87)	0.043	0.131
cDDD as continuous variable	1.01 (0.99-1.02)	0.052	1.01 (1.01-1.02)	0.047	0.130
Vildagliptin					
cDDD <75	0.79 (0.39-1.59)	0.514	0.87 (0.43-1.76)	0.701	0.827
cDDD 75-150	0.71 (0.23-2.21)	0.553	0.80 (0.26-2.50)	0.700	0.827
cDDD >150	1.23 (0.46-3.3)	0.677	1.43 (0.53-3.85)	0.476	0.715
cDDD as continuous variable	1.00 (0.99-1.01)	0.859	1.00 (0.99-1.01)	0.529	0.709

^†^Adjusted p-value obtained through the Bonferroni adjustment test.

## DISCUSSION

We discovered through our executed large population-based study that patients with DM taking a DPP-4 inhibitor did not exhibit a relatively high PD risk. Moreover, we attained this discovery when we analyzed the patients with DM in subgroups defined by the specific DPP-4 inhibitor prescribed, namely sitagliptin, saxagliptin, linagliptin, or vildagliptin. We determined greater age and higher DCSI scores to be linked to increased PD risk. An elevated risk was also discovered for patients with comorbid anxiety.

DPP-4 inhibitor use results in levels of GLP-1 [24, 25] and GIP [25] being elevated, and such elevations may have neuroprotective effects, as was noted in an animal model of PD [26]. However, several challenges must still be overcome. DPP-4 inhibitors are preferred because they do not reach the central nervous system. They are less able to penetrate the blood–brain barrier (BBB) than are GLP-1 agonists [27]. Research has revealed that for patients who receive treatment with DPP-4 inhibitors such as saxagliptin and its primary metabolite [28], linagliptin [29], and vildagliptin [30], these inhibitors can only be detected in the brain in very low levels; thus, scholars have concluded that these compounds are unable to cross the BBB [30]. DPP-4 inhibitors also have several potentially deleterious effects, including that they can lead to a decreased glucagon level, which can result in the suppression of ketosis, lower neuronal tolerance of hypoxia, and higher neuropeptide Y levels, and this may result in vasoconstriction [31]; neuropeptide Y can cause blood flow disorders by inducing various pathophysiological alterations [32]. Furthermore, DPP-4 inhibitors’ inability to cross the BBB may limit the application of most of them. Because different DPP-4 inhibitors may have differing mechanisms of action through which they affect PD development, we performed a drug-by-drug evaluation in our investigation of PD risk in individuals who were diagnosed as having DM and were prescribed a DPP-4 inhibitor.

We noted no increase in PD risk in patients with DM who had a DPP-4 inhibitor cDDD of <75, 75–150, or >150. A significant reduction in PD incidence was observed in 980 patients with DM who were prescribed a DPP-4 inhibitor (including sitagliptin, saxagliptin, and vildagliptin; odds ratio: 0.23, 95% CI: 0.07-0.74) in a previously executed case–control study [33]. However, that study involved a small sample. In a longitudinal cohort study, DM was discovered to be associated with the onset of PD (incidence rate ratio: 0.64, 95% CI: 0.43-0.88) [34]. DPP-4 inhibitors were found in a retrospective study to have a beneficial effect in patients with DM and PD (n = 54); these patients had higher long-term motor performance and greater baseline dopamine transporter availability than did patients with PD but without DM (n = 558) or patients without DM who did not receive a DPP-4 inhibitor (n = 85) did [35].

We noted in our executed study that patients with DM who received sitagliptin did not exhibit a relatively high PD risk, regardless of whether the cumulative dosage was high or low. Sitagliptin can enable recovery of memory deficits by upregulating brain-derived neurotrophic factors, thereby preventing neurodegeneration and dendritic spine loss [36]. Sitagliptin treatment has promise as an approach to preventing the progression of PD because of its antiapoptotic, neurotrophic, neurogenic, and anti-inflammatory activities [37]. However, harmful effects of sitagliptin have been noted in some studies. In a study of rats with DM, tau phosphorylation in the hippocampus was not ameliorated by sitagliptin, which was instead discovered to worsen tau phosphorylation in primary cortical neurons [20]. Although sitagliptin has been suggested by several studies to reduce PD risk [36, 37], any protective effect of this DPP-4 inhibitor may be negated by the tau phosphorylation associated with its long-term use and use at a high cumulative dosage [20]. Nonetheless, scholars have yet to elucidate or clarify the specific mechanism linking sitagliptin use to PD risk; this thus necessitates a large-scale randomized controlled trial to clarify the association and mechanism.

In our study, patients who received saxagliptin did not have an increased PD risk, similar to the result for sitagliptin. Saxagliptin treatment was suggested in an animal study to be a potential beneficial therapy for PD management because it can significantly improve motor function and has antiparkinsonian effects with underlying neurorestorative, neuroprotective, antiapoptotic, anti-inflammatory, and antioxidant mechanisms [18]. By preventing rotenone-induced neurotoxicity, saxagliptin was found to exert a neuroprotective effect by preserving GLP-1, aiding the survival of dopaminergic neurons. Thus, saxagliptin may be suitable for managing PD [18]. The observed effects of saxagliptin are associated with DPP-4 inhibition, increased dopamine synthesis, and reduced neurodegeneration [18]. However, researchers have reported several potentially harmful effects of saxagliptin. In a study in which PD was induced in rats by using 6-hydroxydopamine, no neuroprotective effect or improvement of cognitive or motor deficiencies was discovered upon administration of saxagliptin; furthermore, in the sham group, saxagliptin caused impairment of nonspatial object memory [38]. Associations have been found between DPP-4 inhibitors and the activities of other DPPs, such as DPP-8 and DPP-9 [39]. Several previously executed research works have also found DPP-4 inhibitors to strongly affect the immune system and to strongly inhibit the activities of DPP-8 and DPP-9 [40–42]. Selective DPP-8 and DPP-9 inhibitors were discovered in a rat toxicity study to be associated with severe toxicities leading to an enlarged spleen, multiorgan histopathological changes, and alopecia [43]. Another study reported that after saxagliptin was administered, saxagliptin and its primary metabolite could be detected at only very low levels in the brain [28], indicating that saxagliptin does not cross the BBB [30]. Therefore, saxagliptin’s failure to penetrate the BBB may constrain its neuroprotective effects; moreover, the mechanism underlying saxagliptin’s effects must be further explored before this drug can be tested in clinical trials. Such trials with large samples and long treatment durations are required to determine saxagliptin’s efficacy.

We discovered that patients who received vildagliptin did not have an increased risk of PD. Vildagliptin has been found to have various neuroprotective effects and is promising as a medication for managing PD. An animal study reported that vildagliptin exerts a potential antiparkinsonian effect by inhibiting the RAGE/NF-κB cascade as well as vicious downstream inflammatory, oxidative, and apoptotic cascades [26]. A mouse study revealed that vildagliptin restores 1-methyl-4-phenyl-1,2,3,6-tetrahydropyridine (MPTP)-engendered dopaminergic neurodegeneration in the substantia nigra pars compacta and striatum and, by inhibiting dopaminergic neuronal apoptosis, protects against motor dysfunction caused by MPTP [19]. A direct enzymatic assay revealed that vildagliptin has significant concentration-dependent inhibitory effects on DPP-8 and DPP-9, but that treatment with selective inhibitors of these enzymes results in severe toxicities [43]. In our study, patients who received vildagliptin did not exhibit an increased risk of PD. A possible explanation for this is that vildagliptin cannot readily cross the BBB in humans [30]. Whether and how vildagliptin affects PD risk in humans should be evaluated in further in-depth studies.

Our study also revealed no link between linagliptin use and increased PD risk. Because of the several reported neuroprotective effects of linagliptin, it has promise as a drug for managing PD. A previously executed study indicated that linagliptin’s neuroprotective effect may be linked to its GLP-1-related antioxidant, antiapoptotic, and anti-inflammatory activities [44]. MARK4 plays a role in the development of DM and cancer among other neurodegenerative diseases; linagliptin is a potential inhibitor of MARK4 and may act as a molecule against MARK4-mediated neurodegenerative diseases [45]. In an MPTP-induced PD mouse model, linagliptin regulated microglial polarization and the NLRP3 inflammasome signaling pathway, which led to attenuated neuroinflammation and thus resulted in neuroprotective effects [46]. In our study, the patients who received linagliptin did not exhibit an increased risk of PD. A possible explanation for this is that linagliptin also cannot readily pass the BBB in humans [30]. Linagliptin barely penetrates the brain, mainly because of the action of P-gp [47]. The neuroprotective effects of linagliptin may be limited by this inability to cross the BBB.

Research has reported some positive effects of sitagliptin [48], saxagliptin [18], vildagliptin [26], and linagliptin [49] in animal models. However, in the aforementioned research, these drugs were administered in doses higher than would be typical for humans; therefore, it may be inappropriate to extrapolate the results of these studies to humans. In one study, an absorption–distribution–metabolism–excretion–toxicity analysis revealed that the BBB cannot be penetrated by any DPP-4 inhibitors other than teneligliptin and trelagliptin [50]. Another study reported the same result except that omarigliptin was the DPP-4 inhibitor found to be capable. Therefore, DPP-4 inhibitors may exert effects by raising the levels of GLP-1 [51]. Whether the beneficial effects discovered in animal models would also be present in humans remains unclear; the effectiveness of DPP-4-inhibitor treatment in humans should be evaluated in in-depth research.

In the present study, associations of DPP-4 inhibitor use, higher DCSI score, and older age with elevated PD risk were found. Whether PD develops and progresses is most strongly affected by age [52], and as a person ages normally, the sensitivity of their peripheral insulin receptors decreases [9]. This contributes to a decrease in glucose tolerance with age, and, in humans, insulin resistance often goes hand in hand with age-related glucose intolerance. Nevertheless, the levels of circulating insulin in older people were reported to be similar to those in younger people [53]. Similar to our finding, a previously executed study indicated that PD prevalence increases with an increase in age [54].

We also discovered that among patients diagnosed as having DM, those having comorbid anxiety have a higher PD risk. A systematic review also reported that PD is positively associated with anxiety [55]. Among such patients, anxiety can occur at any disease stage, including the premotor phase. Moreover, in a previously executed study, the PD population was revealed to exhibit higher anxiety when contrasted against the general population [56].

We outline our executed study’s strengths as follows. First, the sample investigated herein was obtained from Taiwan’s total population and thus can be considered representative of the Taiwanese population. The LHID, used as the source of data in the present study, offers a powerful means of conducting research with rich dimensions, and the results of such research can constitute a valuable reference for real-world evidence-based medical research in Taiwan. In addition, a population-based design was employed for the present research, and this design meant that potential selection, prevalent user, and immortal time biases were minimized; such types of bias occur frequently in observational research. Second, we could evaluate PD risk in Taiwanese patients with DM-prescribed DPP-4 inhibitors with sufficient statistical power because of the use of a national database. Third, we conducted a cDDD subgroup analysis to further evaluate the DPP-4 inhibitor–PD risk association.

We also outline our executed study’s limitations as follows. First, the LHID does not contain data on lifestyle factors such as body mass index, physical activity, caffeine intake, alcohol consumption, and smoking. Whether these factors would have influenced our findings due to effects on PD development is unclear [57]. Second, ICD-9-CM and ICD-10-CM codes were the sole basis of PD and comorbidity diagnoses. Nevertheless, the National Health Insurance Administration of Taiwan ensures that diagnoses are accurate by randomly reviewing patients’ charts and interviewing patients. Outlier charges or practices can lead to a hospital being audited, and heavy penalties are imposed for any identified malpractice. The validity and accuracy of the database are ensured by these processes. Third, ICD-9-CM and ICD-10-CM codes indicate a diagnosis but bear no information on the severity of diseases such as DM and PD; subgroup analyses based on disease severity could thus not be conducted. For example, although HbA1c is crucial to hyperglycemia management, no data on HbA1c levels in our enrolled patients with DM were available. Thus, we could not determine whether poor glycemic control was associated with PD incidence.

In conclusion, our derived findings reveal that patients who were diagnosed as having DM and were prescribed a DPP-4 inhibitor did not exhibit a relatively high PD risk during the 3-year period for which they were followed up. Furthermore, patients who were prescribed the DPP-4 inhibitor sitagliptin, saxagliptin, linagliptin, or vildagliptin did not exhibit a relatively high PD risk. We, nevertheless, noted a link of older age and higher DCSI score with a relatively high PD risk. Moreover, we noted the PD risk to be relatively high in patients with DM and comorbid anxiety.
